# Recoding of the stop codon UGA to glycine by a BD1-5/SN-2 bacterium and niche partitioning between Alpha- and Gammaproteobacteria in a tidal sediment microbial community naturally selected in a laboratory chemostat

**DOI:** 10.3389/fmicb.2014.00231

**Published:** 2014-05-16

**Authors:** Anna Hanke, Emmo Hamann, Ritin Sharma, Jeanine S. Geelhoed, Theresa Hargesheimer, Beate Kraft, Volker Meyer, Sabine Lenk, Harald Osmers, Rong Wu, Kofi Makinwa, Robert L. Hettich, Jillian F. Banfield, Halina E. Tegetmeyer, Marc Strous

**Affiliations:** ^1^Microbial Fitness Group, Max Planck Institute for Marine MicrobiologyBremen, Germany; ^2^UT-ORNL Graduate School of Genome Science and Technology, University of TennesseeKnoxville, TN, USA; ^3^Chemical Science Division, Oak Ridge National LaboratoryOak Ridge, TN, USA; ^4^Faculty of Electrical Engineering, Mathematics and Computer Science, Delft University of TechnologyDelft, Netherlands; ^5^Department of Earth and Planetary Science, Department of Environmental Science, Policy, and Management, University of CaliforniaBerkeley, CA, USA; ^6^Center for Biotechnology, University of BielefeldBielefeld, Germany; ^7^Department of Geoscience, University of CalgaryCalgary, AB, Canada

**Keywords:** continuous culture, enrichment, chemostat, *Roseobacter*, *Maritimibacter*, stop codon

## Abstract

Sandy coastal sediments are global hotspots for microbial mineralization of organic matter and denitrification. These sediments are characterized by advective porewater flow, tidal cycling and an active and complex microbial community. Metagenomic sequencing of microbial communities sampled from such sediments showed that potential sulfur oxidizing Gammaproteobacteria and members of the enigmatic BD1-5/SN-2 candidate phylum were abundant *in situ* (>10% and ~2% respectively). By mimicking the dynamic oxic/anoxic environmental conditions of the sediment in a laboratory chemostat, a simplified microbial community was selected from the more complex inoculum. Metagenomics, proteomics and fluorescence *in situ* hybridization showed that this simplified community contained both a potential sulfur oxidizing Gammaproteobacteria (at 24 ± 2% abundance) and a member of the BD1-5/SN-2 candidate phylum (at 7 ± 6% abundance). Despite the abundant supply of organic substrates to the chemostat, proteomic analysis suggested that the selected gammaproteobacterium grew partially autotrophically and performed hydrogen/formate oxidation. The enrichment of a member of the BD1-5/SN-2 candidate phylum enabled, for the first time, direct microscopic observation by fluorescent *in situ* hybridization and the experimental validation of the previously predicted translation of the stop codon UGA into glycine.

## Introduction

The Wadden Sea along the northern European coast is the largest tidal system worldwide and has been a UNESCO world heritage area since 2009. It receives nutrients, mainly in the form of nitrate, phosphate, and silicate from a large catchment area in northern Europe, stimulating growth of algae and other microorganisms in the surface water. Tidal pumping of this water through the permeable sediments of tidal flats leads to the continuous removal of nutrients by highly active indigenous benthic microbes. For example, the measured *in situ* denitrification rates are very high, up to 60 μmol m^−2^ h^−1^ (Kieskamp et al., 1991).

In the past decades the biogeochemistry and the microbial diversity of the Wadden Sea tidal flat have been studied intensively. The microbial community in the upper oxic tidal flat sediments was shown to be dominated by populations of Gammaproteobacteria (Lenk et al., 2011) and Flavobacteria (Llobet-Brossa et al., 1998; Eilers et al., 2001). Potential sulfur oxidizing Gammaproteobacteria make up for an important part (ca. 39.6%) of all Gammaproteobacteria found on Janssand. Within Gammaproteobacteria these bacteria form a large phylogenetic clade that also includes the ubiquitous marine SUP05 cluster (Walsh et al., 2009), many bacterial symbionts of marine invertebrates (e.g., Kleiner et al., 2012) and some cultivated species such as *Sedimenticola selenatireducens* (Narasingarao and Häggblom, 2006), *Dechloromarinus chlorophilus* (Coates and Achenbach, 2004) and *Thiotaurens thiomutagens* (Flood, 2010). Many of these bacteria are known to be facultative aerobic chemolithoautotrophs that can use inorganic electron donors such as sulfide and hydrogen to perform aerobic respiration and denitrification (Walsh et al., 2009; Stewart, 2011). The Flavobacteria on the other hand are known to be involved in the degradation of macromolecules such as polysaccharides (Kirchman, 2002).

To understand the overall function of individual bacterial species in complex natural microbial communities microbiology has traditionally depended on the isolation of target microbes in pure culture. Because such isolation is often unsuccessful, metagenomic genome reconstruction (Tyson et al., 2004; Lo et al., 2007; Wrighton et al., 2012; Castelle et al., 2013) and single cell genomics (Kalisky and Quake, 2011; Blainey, 2013; Rinke et al., 2013) have been used to unravel the metabolism of key community members without their isolation. Both approaches can yield near-complete (Tyson et al., 2004; Baker et al., 2010; Wrighton et al., 2012) and, in some cases, complete genomes (e.g., Albertsen et al., 2013; Castelle et al., 2013; Rinke et al., 2013). However, sequencing methods alone lack the ability to probe metabolic function and provide limited insight into microbial interactions.

Both with conventional pure cultures and single cell methods, the target microorganism is, literally, isolated from its natural context and it is often difficult to understand the ecological niche of the isolated microorganism. For this, it would be ideal to study the organism in the context of its natural habitat. Because of the dynamics and complexity of natural communities this is generally not straightforward.

Engineering of a simplified natural ecosystem in the laboratory can enable the study of uncultivated bacteria in the context of a simplified microbial community (e.g., Kartal and Strous, 2008; Belnap et al., 2009). Such a simplified microbial community will assemble spontaneously from a more complex inoculum by natural selection under the conditions applied. In combination with metagenomic genome reconstruction, proteomic and metabolomic approaches this allows for characterization of overall community metabolic function, interactions, and provides a route to unravel the contribution of each of the individual members. Once an engineered system is available, key environmental factors that define the ecological niche of selected populations can be identified by manipulating the applied conditions. Such manipulation is not possible when natural ecosystems are studied directly.

To achieve a significant substrate turnover at low, near *in situ* substrate concentrations, habitat engineering depends on continuous culture cultivation, e.g., a chemostat. The low substrate concentrations favors the selection of K-strategists (“oligotrophs”) relative to r-strategists (“microbial weeds”) (Andrews and Harris, 1986). Further, in a continuous culture the applied conditions are stable (or dynamic, if desired) and reproducible for an indefinite amount of time.

In the present study we simulated the environmental conditions of a tidal flat sediment in a simplified form. The most significant difference between the simulated and the natural environment was that in the natural habitat, microbes grow as thin biofilms on sand grains, whereas in the simulated habitat, cells grew in suspension. Nitrate and nitrite were supplied as the main electron acceptors and oxygen was supplied twice daily, mimicking tidal cycling. The carbon and energy source consisted of a mixture of glucose, amino acids and acetate, in a ratio similar to decaying biomass, the main carbon and energy source *in situ*. After 23 days the resulting community was shown to be dominated by representatives of phylogenetic clades also detected in the tidal flat sediments, including a population of potential sulfur oxidizing Gammaproteobacteria and a member of the enigmatic bacterial BD1-5/SN-2 clade which lacks cultivated representatives and was previously predicted to translate the stop codon UGA into glycine. Proteomic analysis of the simplified microbial community enabled the experimental validation of this prediction.

## Materials and methods

### Sampling and inoculation

Sediment samples were taken from an intertidal flat in the central German Wadden Sea known as “Janssand” located south of the Eastern Friesian Island Spiekeroog (N: 053° 44′ 151″/E: 007° 41′ 945″). For direct metagenomic sequencing one sample was collected on October 24 2009 (0–5 cm depth), and three samples on March 23 2010 (0–2 cm depth). To assess small scale differences, the three March samples were taken form locations approximately 3 m apart. To analyze sequencing bias, two of the three March samples were pooled, and the pool was divided into two samples, named Mar10/1a and Mar10/1b, to be sequenced in separate sequencing runs. Sediment samples were stored at −20°C in 50 ml falcon tubes. For inoculation sampling was conducted at low tide in May 2011 (15°C, overcast). Using a trowel, sediment from the upper 2 cm layer of Janssand was shovelled into a cooling box and kept on ice during its 5 h transport to the lab. It was diluted with artificially prepared seawater (33.4 g/l salt, Red Sea, Somerset, UK; in Milli-Q water) in a ratio of 1:1 and mixed for 3 min using a drilling machine (PSB 850-2 RE, 850 Watt, Bosch, Stuttgart, Germany) with a cement mixer. The turbid, sand free supernatant (the “cell extract”) was filled in 800 ml portions into 1 l transfusion bottles (Ochs Glasgerätebau, Bovenden/Lenglern, Germany) and pH was set to 8.1–8.4 with 1 M NaOH solution. The cell extract was made anoxic by alternately applying vacuum to 0.3 bar and argon to 1.2 bar, 3 times each. Each bottle was supplemented with NaNO_3_ stock solution to reach a final concentration of 0.1 mM serving as electron acceptor. 50 mg/l cycloheximide (AppliChem, Darmstadt, Germany) was added and the cell extract was incubated at 4°C for 24 h to kill predatory eukaryotic organisms. After that, the cell extract was used for inoculation of the continuous culture.

### Denitrification rates before and after sediment extraction

For a direct comparison, denitrification rates were determined for fresh sediment and cell extract (the inoculum, see above). Batch incubations were conducted as described by Würgler Hansen et al., 2000 with minor modifications: Fresh sediment samples and cell extract (see above) were incubated in triplicates in 400 ml nearly gas tight plastic bags (manufactured by sealing from transparent 3-layer PA/PE tubular bags BST-090; Rolf Bayer Vakuumverpackung GmbH, Veitsbronn, Germany). Manufactured glass windings with flat flanges and flanged sockets (Ochs Glasgerätebau) served as openings, tightened with GL14 screw caps with holes (Ochs Glasgerätebau), and closed with natural rubber stoppers (D10, Ochs Glasgerätebau). Air exclusion was achieved by additionally using NBR o-rings (Franz Gottwald GmbH and Co KG, Bremen, Germany) and viton flat gaskets (Rubber BV, Hilversum, The Netherlands). After filling the bags with either 400 ml cell extract or with a mix of sediment and sea water (ratio 1:1) they were sealed with a bag sealer (Polystar 110 GE, Rische + Herfurth GmbH, Hamburg, Germany). The sealed bags were amended with Na^15^NO_3_ (0.2 mM final concentration, Sigma Aldrich, Munich, Germany) and with NH_4_Cl (1 mM final concentration; to prevent NO_3_ consumption via assimilation; Roth, Karlsruhe, Germany), manually mixed, and incubated in the dark at room temperature. Sampling of the bags was conducted directly after filling and 4 more times in 90 min intervals. Without leaving a headspace the liquid samples were filled into 3.9 ml soda glass vials (Exetainer®, Labco Ltd., Lampeter Ceredigion, UK) pre-filled with HgCl_2_ (saturated in final volume; Roth) to stop all biological activity. A 1.5 ml helium headspace was set to the vials. Headspace samples were analyzed with a GC/IRMS (Fisons VG Optima, MasCom, Bremen, Germany) to determine the N-isotopic composition of N_2_ gas produced during the incubations.

### Species richness before and after sediment extraction

Automated ribosomal intergenic spacer analysis (ARISA) was performed to quantify the loss of species during sediment extraction. DNA extraction prior to ARISA was according to Zhou et al. (1996). The ARISA procedure was conducted as described by Ramette (2009).

### Medium composition

The cultures were continuously supplied with anoxic, sterile liquid medium. Composition of the medium is shown in Table 1. Nitrate (1 mM) and nitrite (20 mM) served as electron acceptors while a defined mixture of different carbon compounds (30.5 C-mM) was supplied as electron donor. The nutrient concentration in the medium was increased within the first 14 days of the incubation from 5 mM nitrite (stable nitrite:nitrate:carbon ratio as mentioned in Table 1) up to 20 mM. However, nutrient concentrations in the culture were generally in the low micromolar range because the medium entered the chemostat dropwise.

**Table 1:** **Medium composition for continuous cultures**.

**Substance class**	**Substance**	**Concentration**	**Supplier**
	**Red sea salt**	33.4 g/l	Red sea
**NUTRIENTS**		**mM**	
	NaNO_3_	1	Roth
	NaNO_2_	20	Roth
	Carbon (see below)	30.5	–
**TRACE ELEMENTS**		**μM**	
	KH_2_PO_4_	500	Roth
	FeSO_4_*7H_2_O	10.755	Roth
	CuCl_2_	1.256	Roth
	ZnCl_2_	0.051	Roth
	MnCl_2_*4H_2_O	0.051	AppliChem
	H_3_BO_3_	0.097	Roth
	CoCl_2_*6H_2_O	0.05	Roth
	NiCl_2_*6H_2_O	0.011	Roth
	Na_2_MoO_4_*2H_2_O	0.011	Roth
	AlCl_3_*6H_2_O	0.01	AppliChem
**CARBON COMPOUNDS**		**%Carbon**	
	Glucose	44.1	AppliChem
	Acetate	7.6	AppliChem
	Amino acids (see below)	48.3	–
**AMINO ACIDS**		**%Carbon**	–
	Glutamic acid	10.7	AppliChem
	Aspartic acid	11.7	Roth
	Alanine	8.5	AppliChem
	Serine	4.6	AppliChem
	Tyrosine	8.9	AppliChem
	Histidine	1.4	AppliChem
	Methionine	2.4	AppliChem

Note that the actual substrate concentrations inside the chemostat were much lower because the influent medium was diluted dropwise into the culture vessel.

### Continuous cultivation

**Figure 1:**
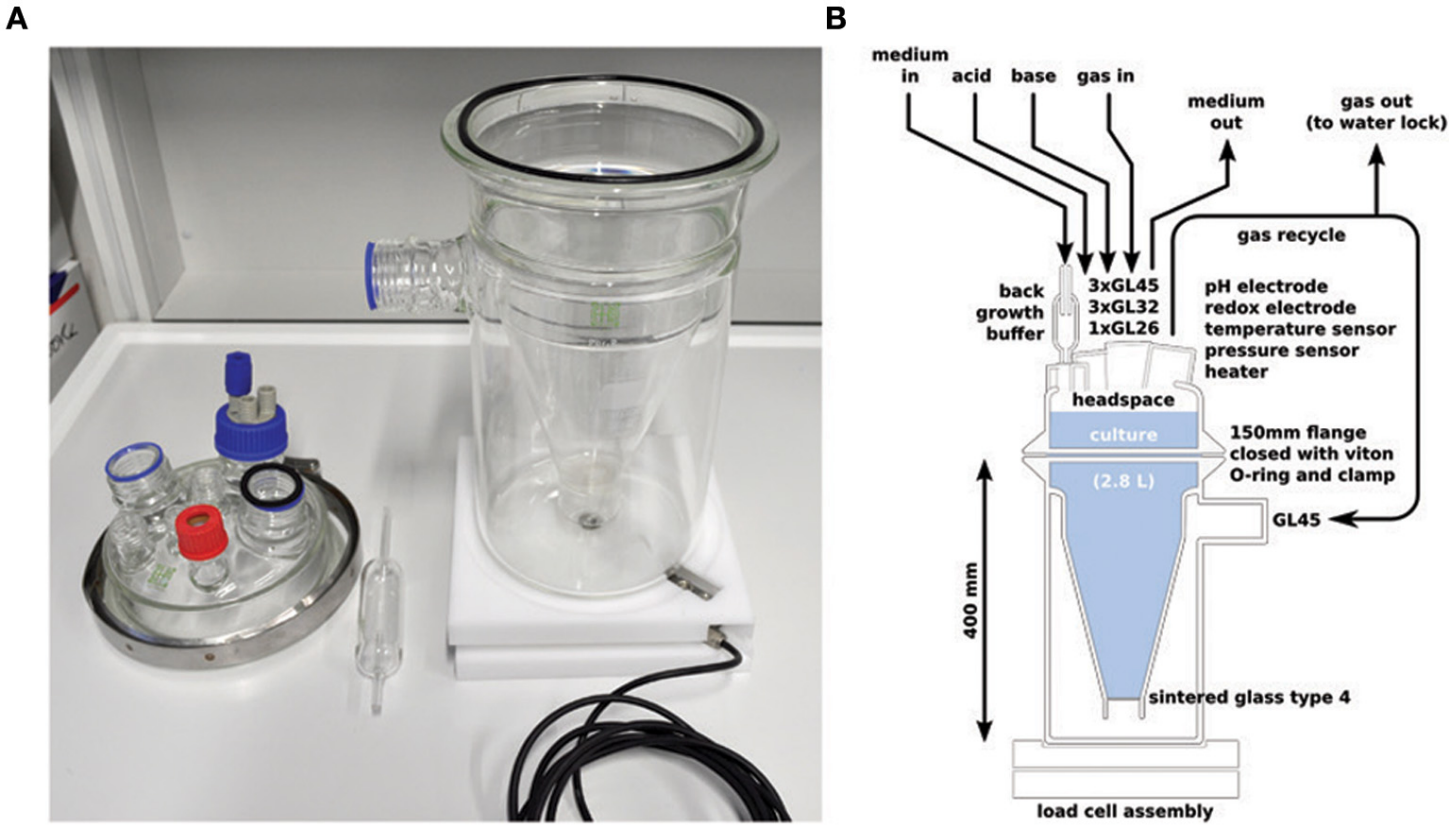
**The chemostat vessel used, photo (A) and schematic (B).** All additions and withdrawals were made from the top of the vessel. The vessel was mixed by recycling gas from the headspace to the bottom via a sintered glass filter. The culture volume was measured via the vessel weight by a load cell mounted underneath the vessel. Medium was added via a glass back growth buffer to prevent contamination of the influent medium tubing.

The custom culture vessel (Ochs Glasgerätebau) consisted of a so called “Kluyver flask” (an inverted Erlenmeyer with a sintered glass disk inserted in its neck) fixed inside a beaker. The bottom of the Erlenmeyer was removed and replaced by a lid with standard Teflon Schott connectors for inserting sensors and tubing see Figure 1. The flask content was mixed by recycling gas (4 l min^−1^; 620S peristaltic pump, Watson Marlow, Wilmington, MA, USA) from the headspace of the flask via the beaker and the sintered glass disk into the culture. Recycled gas was continuously amended by 10 ml min^−1^ argon with a mass flow controller (Alicat Scientific, Tucson, AZ, USA). Part of the gas left the culture via a water lock to maintain a constant overpressure of 13 ± 5 mbar. The liquid volume of the culture was 2.8 l and the gas volume of the combined headspace and beaker was 3.08 l. Medium was pumped into the vessel continuously (0.7 ml min^−1^, dilution rate 0.36 day^−1^) via a glass back growth buffer (Figure 1) to prevent colonization of the medium supply tubing by the enriched bacteria. Culture liquid was continuously removed by a second peristaltic pump controlled by a weight sensor (load cell, mounted underneath the culture) to keep the volume constant. The pH was automatically controlled to 8.0 ± 0.05 with 0.2 M NaOH and 0.25 M HCl solutions. To simulate the periodically aerobic/anaerobic conditions associated with a tidal system, from day 9–28 pure oxygen (0.2 ml min^−1^) was added for 120 min every 12 h. The gas recycling of the cultivation vessel led to a rapid exchange of oxygen from the gas to the liquid. The temperature was controlled at 25°C with a resistive heater controlled by a temperature sensor (see below). Both the heater and the temperature sensor were mounted into NMR tubes with help of a thermal compound (Amasan Wärmeleitpaste T12, Jürgen Armack GmbH, Norderstedt, Germany) so that no metal contacted the culture fluid directly.

The continuous culture was controlled by an embedded National Instruments compactRIO real time power PC (cRIO-9014, 128 Mb RAM, 2 GB disc on chip non-volatile storage) combined with an 8-slot Virtex-5 FPGA chassis (cRIO-9112) that interfaced the input/output modules. Seven modules were added to the chassis and they consisted of: (1) a 16 channel (differential) 16 bit 10 V 250 kS/s analogue to digital converter (NI9205), (2) an 8 channel 16 bit 20 mA 200 kS/s analogue to digital converter (NI9203), (3) a 4 channel 24 bit 60 V 100 S/s analogue to digital converter (NI9219), (4) a 16 channel 16 bit 10 V 25 kS/s digital to analogue converter (NI9264), (5) a 32 channel 5 V TTL digital input/output module (NI9403), (6) a high speed (1 μs response time) 8 channel 24 V digital output module (NI9474) and (7) a 8 channel 60 V digital relay module (NI9485). These components were built into a standard 19 inch rack together with a power supply and the electronics for signal conditioning (see below). Sensor signals were acquired and digitized by the analogue to digital converters. Process control (proportional-integral, integration time 2 min) was implemented in hardware logic on the Virtex 5 FPGA and was completely independent of the microprocessor unit for maximum operational stability. Control actions were converted to analogue signals by the digital to analog converters or via pulse width modulation to the digital output modules. The real time microprocessor unit was only used for data logging on the solid state hard disc, communication (ethernet, TCP/IP) with an external computer and for creating dynamic conditions inside the culture vessel in an automated way.

The pH was measured with combined pH electrodes (HA405-DPA-SC-S8/120; Mettler Toledo, Giessen, Germany). Redox potential was measured with Mettler Toledo redox electrodes (Pt4805-60-PA-S8/120). The signals of both redox and pH electrodes were conditioned with Picos 1201PH conditioners. These conditioners converted the signal into a 4–20 mA format and interfaced with the NI9203 module of the controller. The overpressure in the headspace of the culture vessels was measured with PA-23S pressure transducers (Comer, Bologna, Italy) that also provided a 4–20 mA signal for the same module. The liquid volume inside the culture vessel was measured gravitometrically with a load cell (LSH-10kg-C3, combined error <0.017%, AE sensors, Dordrecht, the Netherlands) underneath the culture vessel. The signal was conditioned with an ICA3 signal conditioner (same supplier, output signal 0–10 V, digitized by module NI9205) built inside the 19 inch rack. Temperature was measured with temperature sensors consisting of two 45016 SP20725 thermistors (YSI, Yellow Springs, Ohio, USA) per sensor. Both thermistors were part of a Wheatstone bridge and measurement noise was filtered and the signal was digitized by a custom analogue to digital converter as previously described (Wu et al., 2012). The resulting bitstream interfaced with module NI9403. With this setup the accuracy of the temperature measurement was around 1–10 μK. For less precise but more flexible measurements a pt1000 sensor was also used. The chemical composition of the headspace gas inside the culture was monitored periodically with a GAM400 online mass spectrometer (InProcess, Bremen, Germany) during a complete tidal cycle.

Pumping of fluids (medium, effluent, alkaline and acid solutions for pH control) was performed with OEM peristaltic variable speed pumps (IS3124A) and controllers (IS3186) obtained from Ismatec (Wertheim, Germany) interfacing with module NI9264. Four of these pumps and controllers were built into a single 19 inch rack. Gases were supplied with mass flow controllers (Alicat Scientific) controlled by module NI9264. The feedback signal from the controllers (the actual gas flow) was acquired with module NI9205. Heating was performed with custom cartridge resistors (4 mm diameter, 60 mm long, 20 W at 24 V) obtained from Türk Hillinger (Tuttlingen, Germany). These interfaced (with pulse width modulation at 20 kHz) with module NI9474.

### Wet chemical analyses

Nitrite was measured spectrophotometrically according to Bendschneider and Robinson (1952). Nitrate concentrations were calculated by subtraction of nitrite concentrations (see above) from the total NO_x_ concentration determined with an NO/NO_x_ analyzer (2 μM detection limit; CLD 66 S, Eco Physics, München, Germany). Samples were injected into the reaction chamber connected upstream to the analyzer. At 90°C with acid VCl_3_ (0.1 M), both NO^−^_3_ and NO^−^_2_, were reduced to NO which was measured by the chemiluminescence detector. Ammonia in the culture liquid was measured according to Solorzano (1969). Protein was measured spectrophotometrically according to Lowry et al. (1951).

### DNA sequencing for metagenomics

For the sediment metagenome 500 ng of purified DNA per sample were used for the preparation of sequencing libraries according to the “Rapid Library Preparation Method Manual” (October 2009/Rev. January 2010) provided by Roche. Two GS FLX Titanium sequencing runs were performed with each of the four libraries loaded on half a sequencing picotiterplate. Samples Oct09 and Mar10/1a were sequenced in the same run and sample Mar10/1b was sequenced together with sample Mar10/2.

For the chemostat metagenome,1 μg of purified DNA per sample was used for the preparation of sequencing libraries according to the “Rapid Library Preparation Method Manual” (May 2011) for GS FLX+ Series provided by Roche. The sample was sequenced on a quarter picotiterplate of a GS FLX+ Titanium sequencing run. Compared to the previous version (Titanium chemistry), the obtained reads were long (average read length 671 nt) but fewer reads were obtained (168 169/quarter plate). IlluminaMiSeq sequencing was carried out as follows: 2 μg of purified DNA were used for generating a barcoded PCR-free TruSeq® illumina sequencing library according to the “TruSeq® DNA PCR-Free Sample Preparation Guide” for a median insert size of 550 bp. The library was sequenced on 3% of a flow cell in a paired end run (2 × 255 bases) on a MiSeq instrument. 315 246 read pairs were sequenced, yielding approx. 160 MB sequencing output after quality trimming.

### *In silico* computational procedures

The metagenomic reads of the *in situ* metagenomes were screened for fragments of 16S rDNA genes with six hidden markov models (Eddy, 2011) trained with forward and reverse-complemented eukaryotic, archaeal and bacterial reference 16S rDNA genes from the Silva database, release 12 (http://www.arb-silva.de/). Out of 2511392 reads (average length 394 bp), 862 reads encoding a partial 16S rDNA gene were so identified.These fragments were classified with blastn (Camacho et al., 2009) and the Silva database.

Assembly of the metagenomic reads from the chemostat was carried out (after filtering the reads for quality and removing tags) with the GS De Novo Assembler 2.8 (454 Life Sciences, Branford, CT, USA) using the default settings for genomic DNA. Assembled contigs were binned based on tetranucleotide compositions combined with interpolated Markov models (IMMs) with the Metawatt binner (Strous et al., 2012); briefly, four bins were created based on IMMs trained with tetranucleotide bins “high_bin_0,” “low_bin_1,” “low_bin_2,” and “low_bin_4.” After that the contigs in “low_bin_2” were separated into two bins with a sequencing coverage cutoff of 7x. Per contig sequencing coverage was estimated by mapping the reads to the assembled contigs with bowtie2 (Langmead and Salzberg, 2012) and coverage and bin size were used to estimate the abundance of each binned population. Next, redundant sequence information per bin was identified by BLASTn (Camacho et al., 2009; “internal overlap” in Table 3). Transfer-RNAs were identified with Aragorn (Laslett and Canback, 2004). Genome completeness was estimated for each bin by representation of 139 conserved genes as described by Campbell et al. (2013). The contigs of each bin were annotated separately with Prokka (http://vicbioinformatics.com). Prediction of the alternate genetic code of the BD1-5/SN-2 bacterium was performed as described by Dutilh et al. (2011) and the alternate code was implemented in Prodigal (Hyatt et al., 2010) to enable open reading frame (ORF) prediction and annotation. Full length 16S rRNA gene sequences were obtained by searching the assembled contigs with a custom hidden Markov model (Eddy, 2011) trained with representative 16S rRNA gene sequences from the SILVA database (Quast et al., 2013) and, independently, by iterative read mapping with EMIRGE (Miller et al., 2011). Alignment of 16S rRNA gene sequences was performed with MAFFT (Katoh et al., 2002; high accuracy options—maxiterate 1000—localpair) and a phylogenetic tree was calculated by approximate maximum likelihood with FastTree2 (Price et al., 2010) after applying a 50% conservation filter.

### Proteomics

Based on the protein estimation results from cultures, an aliquot corresponding to 300 μg total protein was used for proteomics sample preparation via the Filter-aided Sample Prep method (FASP) (Wiśniewski et al., 2009). To the 82 μL of sample in an eppendorf tube, 30 μL of HPLC grade water, 30 μL of 10% SDS and 8 μL of 1 M DTT were added. The tube was then boiled at 95°C for 10 min. The sample was cooled to room temperature, and the crude lysate was put on top of a 30 kDa molecular-weight cut-off (MWCO) filter provided with FASP Kits (Expedeon Inc., San Diego, CA, USA). The kits were operated in the standard manner specified for handling GELFrEE fractions. Briefly, the lysed sample was first washed with 200 μL of 8 M urea in 100 mM Tris-HCl (pH 8.5) at 14,000 g for 25 min. The step was repeated twice. Following urea washes, the proteins were alkylated with IAA treatment by incubation in dark for 30 min. Then, the sample was washed three times with 100 μL of 50 mM ammonium bicarbonate solution by centrifuging at 14,000 g for 10 min. Protein digestion was carried out first for 4 h at 37°C using trypsin (Promega) in 1:20 protease to protein ratio. A second aliquot of trypsin was added following first 4 h and sample was incubated at 37°C for an overnight digestion. Peptides then were collected in a fresh tube after washing the filter with two washes of 50 mM ammonium bicarbonate and final addition of 0.5 M NaCl and spinning at 14 000 g. The pH of resulting peptides solution was adjusted to <3 by addition of formic acid.

Approximately 25 μg of peptides were pressure-loaded onto an integrated, self-packed 3 cm Reverse Phase(RP) resin (Aqua, 300 Å pore size, Phenomenex, Torrance, CA, USA) and 3 cm Strong Cation Exchange (SCX) resin in a 150 μm inner diameter fused silica back column. The peptides were desalted on the column by washing from solvent A (95% HPLC H_2_O, 5% AcN, 0.1% Formic acid) to solvent B (30% HPLC H_2_O, 70% AcN, 0.1% Formic acid) 3 times over a period of 25 min. The desalted back column was connected to a 15 cm-long 100 μm-I.D. C–18 RP resin PicoFrit column (New Objective, Wobum, MA, USA) and placed in line with a U3000 quaternary HPLC (Dionex, San Francisco, CA, USA). The SCX-RP LC separation was carried out by eleven salt pulses with increasing concentrations of 500 mM ammonium acetate solution. Each of the first ten salt pulses was followed with 120 min RP gradient from 100% solvent A to 50% solvent B, while the last salt pulse used 150 min RP gradient from 100% solvent A to 100% solvent B. The LC eluent from the front column was directly nanosprayed into an LTQ-Orbitrap Elite mass spectrometer (Thermo Scientific). The mass spectrometer was operated in a data-dependent mode under the control of Xcalibur software (Thermo Scientific). The following parameters were used for the data-dependent acquisition: collision induced dissociation was carried out for top 20 parent ions in the ion trap following a full scan in the Orbitrap at 30 000 resolution, a 0.5 m/z isolation width, 35% collision energy was used for fragmentation; and a dynamic exclusion repeat count of 1 with duration of 30 s. The raw MS/MS data was searched using MyriMatch v2.1 (Tabb et al., 2007) against a predicted protein database (28,627 sequences with bin E translated thrice: UGA as STOP codon, UGA coding for glycine, UGA coding for tryptophan) constructed from metagenome assembly, along with common contaminants (44 sequences) and reverse sequences. A second search was performed using MyriMatch v2.1 against a predicted database same as before, with the only difference being that the binE was translated 20 times with UGA coding for all 20 amino acids as well as UGA acting as a STOP codon. A fixed modification of +57.0214 Da for carbamidomethylation of cysteine and a +16 Da modification for oxidation of methionine and a +43 Da modification for N-terminal carbamylation were included as dynamic modifications in the search parameters. Identified peptides were then filtered at <1% peptide level FDR and assembled into proteins (minimum of two peptides per protein) by IDPicker 3 (Ma et al., 2009). (For more information on search settings see supplementary SOM Table 5). For all bins, two replicate samples were used to calculate average peptide coverages for every predicted and annotated ORF and, finally, average peptide coverages for specific metabolic pathways.

### Catalyzed amplified reporter deposition fluorescence *in situ* hybridization (CARD-FISH)

CARD-FISH was conducted according to Thiele et al. (2011) with minor modifications. The probes applied in FISH targeted the most dominant classes and phyla, respectively, detected in the enrichment (Table 2).

**Table 2:** **Oligonucleotide probes used for FISH analyses in this study**.

**Probe**	**FA(%)**	**Target**	**Sequence(5′ → 3′)**	**References**
BD1207	40	BD1-5 member	AGCCCCAGACGTAAAAGC	This study
Gam42a	35	Gammaproteobacteria	GCCTTCCCACATCGTTT	Manz et al., 1992
Bet42a unl.	35	Betaproteobacteria	GCCTTCCCACTTCGTTT	Manz et al., 1992
Alf968	35	Alphaproteobacteria	GGTAAGGTTCTGCGCGTT	Neef, 1997
Eub338	35	Eubacteria	GCTGCCTCCCGTAGGAGT	Amann et al., 1990
Eub338-II	35	Eubacteria	GCAGCCACCCGTAGGTGT	Daims et al., 1999
Eub338-III	35	Eubacteria	GCTGCCACCCGTAGGTGT	Daims et al., 1999
Non338	35	Negative control	ACTCCTACGGGAGGCAGC	Wallner et al., 1993

unl, unlabeled (competitor to Gam42a); FA, formamide concentration (v/v) in the hybridization buffer.

The novel oligonucleotide probe BD1207 targeting the 16S rRNA of contig01521 (BD1-5/SN-2) was designed using the probe design tool of ARB and the SILVA SSU Ref database. Initial hybridization with the newly designed probe was performed at low stringency using a formamide concentration of 0 and 10% in order to check for positive signals of the targeted population in the bioreactor. Signals of probe BD1207 were considered to be significant as cells were detected uniformly over the filter and counts exceed signals of the negative control probe NON338. The hybridization stringency of the novel probe BD1207 was optimized by a series of hybridizations at increasing formamide concentrations (10–60% in steps of 5%) following determination of signal cut-off (i.e., decrease in signal intensity). The optimal formamide concentration (here 40%) was the highest concentration before signal cut-off occurred.

### Data submission

The four metagenomes obtained directly from the sediment were submitted to the sequence read archive (http://trace.ncbi.nlm.nih.gov/Traces/sra/) under the bioproject PRJNA174601 (Accession: SRP015924). The sample Accession numbers are SRS365699, SRS365698, SRS365700 and SRS365701. The 454 and Illumina sequencing data sets obtained from the chemostat were submitted to the sequence read archive (http://www.ncbi.nlm.nih.gov/Traces/sra/), and the assembled contigs were submitted as metagenome sequencing project to the whole genome shotgun submission portal (https://submit.ncbi.nlm.nih.gov/subs/wgs/). Both reads and contigs are accessible under the BioProject PRJNA209200 (Accession: SRP026254), and the BioSample SAMN02211837 (Accession: SRS451311). The Whole Genome Shotgun project has been submitted to the DDBJ/EMBL/GenBank databases under accession No. ATLU00000000. The version described in this paper is version ATLU01000000.

## Results

After extracting the cells from sediment samples collected from the tidal flat, the potential denitrification rate and community composition of the cell suspension was determined and compared it to the original sediment before extraction. It was found that 30% of the potential denitrification rate (8.4 μmol l^−1^d^−1^ vs. 28.7 μmol l^−1^d^−1^) and 41% of the OTUs (40 out of 97) were recovered, as shown by incubation with ^15^N-nitrate and ARISA, respectively.

The cell suspension was incubated under dynamically oxic/anoxic conditions favoring aerobic respiration and denitrification. It was continuously supplied with medium containing glucose, amino acids, and acetate as carbon sources (see Table 1). The composition of carbon sources was close to the composition of decaying biomass (in terms of its monomers), the prevalent carbon and energy source *in situ*.

**Figure 2:**
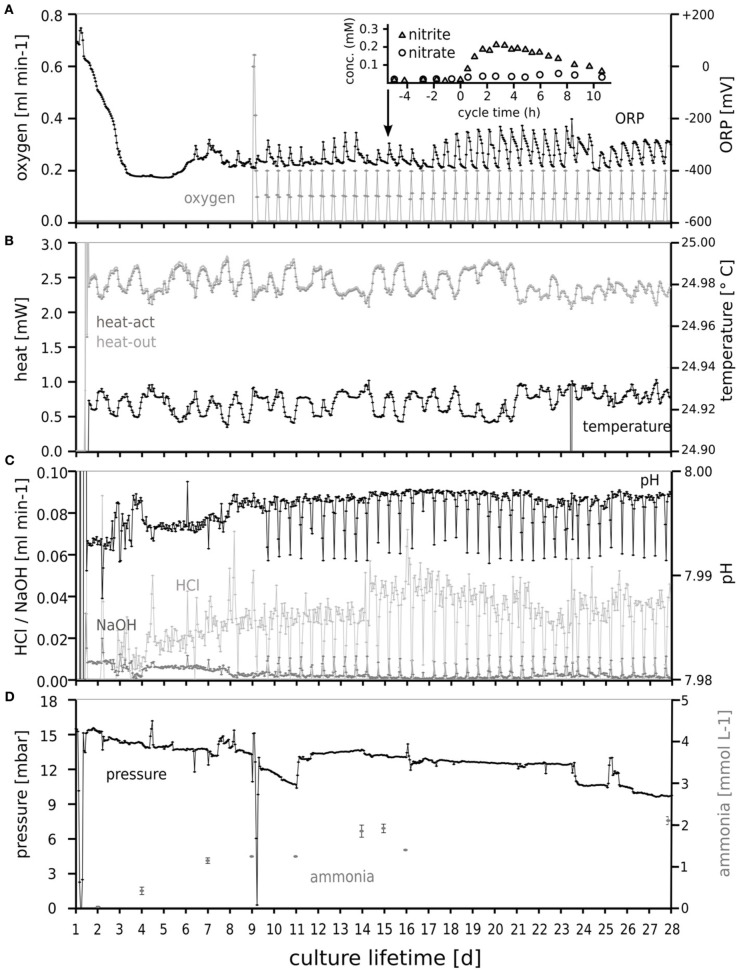
**Oxygen dosage (A), temperature and heater power (B), and pH control (C) enabling dynamic “tidal” conditions for selection of the microbial community in the chemostat during 28 days.** The response of the culture to the intermittent oxygen supply is clearly visible in the redox sensor (**A**, ORP) response. The maintenance of several mbar overpressure **(D)** indicated the absence of undesired air leaks. The ammonium concentration in the culture vessel (measured off-line) is also shown in **(D)**. The response of the nitrite and nitrate concentrations to tidal cycling is shown in the inset of **(A)** for day 15.

The tidal oxic/anoxic cycling was clearly visible in the signal of the redox electrode (Figure 2). During oxygen supply a slight decrease in pH was observed. Over time, pressure decreased slowly from 15 to 9 mbar caused by evaporation of water from the water lock which prevented back-flow of atmospheric gases into the culture. Intermittent steep pressure decreases (Figure 2) resulted from sampling and maintenance operations. Nitrate and nitrite were consumed almost completely and responded to tidal cycling, as shown for day 15 (inset). Accumulation of nitrite and nitrate during tidal cycling attenuated with time. Apparently, the microbial community adapted to the periodic oxygen supply, leading to less nitrite accumulation.

Protein concentrations were measured to determine the overall biomass growth yield. The fraction of assimilated carbon ranged from 19 to 25% of carbon delivered. Ammonia was produced by the culture and its concentration (mM) was consistent with the ammonification of the supplied amino acids.

**Figure 3:**
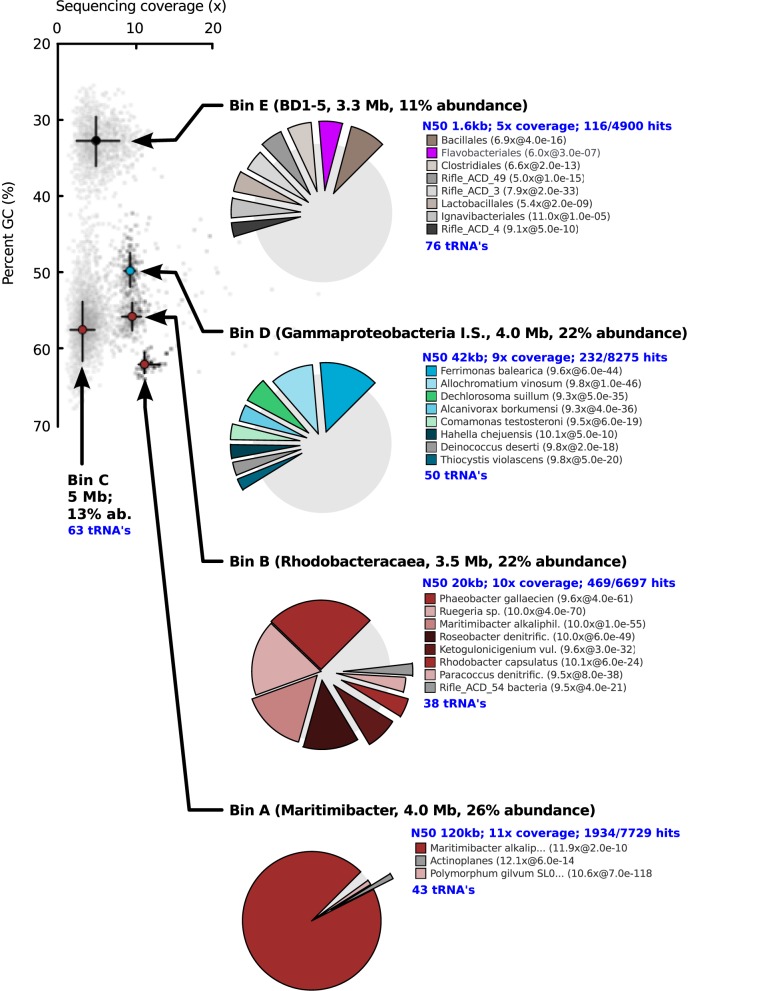
**GC vs. coverage plot showing scattering of the contigs into distinct “clouds,” each associated with a different bin.** Bins were obtained independent of coverage data with a combination of tetranucleotide compositional analysis and interpolated markov modeling. The distribution of best blast hits over reference organisms is shown for four of the five bins. For C, the distribution was very similar to that of bin B. Because no phylogenetically close reference organisms were present in the database for Bin D and E, many contigs of these bins did not give a blast hit.

**Figure 4:**
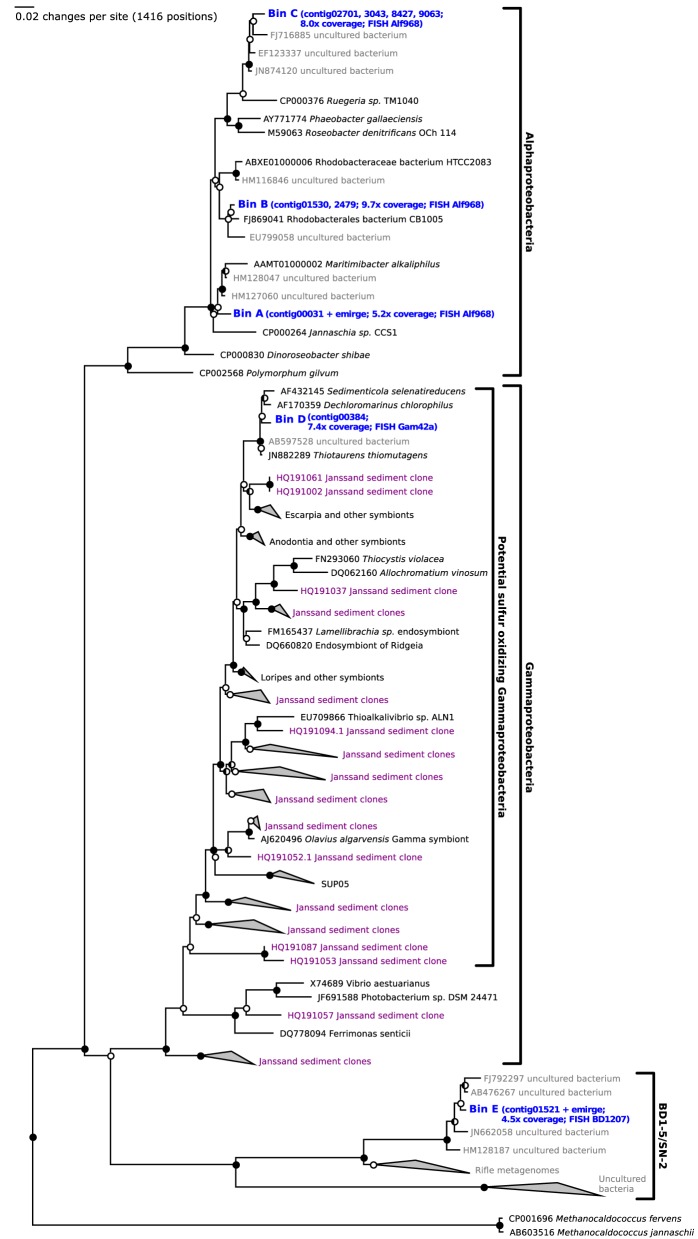
**Phylogenetic analysis of the full length ribosomal 16S rRNA genes reconstructed from the metagenome reads sampled after 23 days of selection (blue).** Closed circles indicate bootstrap values of >95%, half open circles indicate bootstrap values of >90%. Potential sulfur oxidizing Gammaproteobacteria previously detected by Lenk et al. (2011) are shown in purple.

After 14 days of stable oxic/anoxic cycling (23 days after inoculation) the enriched microbial community was characterized by metagenomic sequencing and proteomic analysis. The N50 contig length of the assembly was 4.1 kb at 5.8x sequencing coverage and in total 13.2 Mb of unique sequence data was assembled. Three full length 16S rRNA genes were detected in the *de novo* assembly as well as several fragments that were assigned to *Roseobacter* populations; these were combined into 2 additional full length genes by alignment to reference sequences of closely related bacteria. In parallel, iterative read mapping yielded two full length 16S rRNA genes that were >97% identical to two of the assembled ones. By binning of contigs based on tetranucleotide frequencies five bins were generated, each with a distinct phylogenetic profile that aligned well with the recovered 16S rRNA gene sequences. Figure 3 visualizes the position of each bin on the GC vs. coverage plot of the assembly as well as the per-bin phylogenetic profile calculated. Figure 4 shows a phylogenetic tree of the most important 16S rRNA genes recovered and their assignment to the bins. Based on read mapping, the binned populations were estimated to make up approximately 93% of the overall community.

Table 3 summarizes the taxonomic assignment of the bins, estimated genome sizes and other properties. The most abundant population in the culture (corresponding to bin A) belonged to the genus *Maritimibacter* (Alphaproteobacteria). Bins B and C corresponded to two different *Roseobacter* populations, bin D to a potential sulfur oxidizing Gammaproteobacterium and bin E to a member of the enigmatic bacterial BD1-5/SN-2 clade which lacks cultivated representatives. Based on the presence of conserved genes, each of the bins apparently contained a nearly complete genome sequence of the associated population.

**Table 3:** **Characteristics and metabolism of the binnable populations at *t* = 23 days (n.d. = not detected)**.

**Bin**		**A**	**B**	**C**	**D**	**E**
**Taxonomic clade**		**Maritimi-bacter**	**Roseo-bacter- clade**	**Roseo-bacter- clade**	**Gamma-proteo-bacteria I.S.**	**BD1-5/ SN-2**
Abundance (binning)	(%)	25.9	21.7	12.5	22.3	11.2
Abundance (proteome)	(%)	32.9	25.9	13.2	25.2	2.8
Abundance (*in situ*)	(%)	0.1	2.1	2.1	12.1	1.9
GC content	(%)	62.3	55.9	56.9	50.2	33.2
Bin size	(Mb)	3.7	3.5	5.0	4.0	3.3
Internal overlap	(%)	2.7	1	12.2	3.2	37.2
Number of tRNAs	(#)	43	38	63	50	76
Genome completeness	(#/#)	122/139	133/139	105/139	135/139	109/139
Estimated genome size	(Mb)	3.6	3.5	4.4	3.9	2.1
Number of contigs	(#)	163	429	4597	248	2747
N50 contig length	(kb)	120	20	1.4	42	1.6
**Expressed subsystems**		**#Expressed proteins detected (peptide coverage)**				
Ribosomal proteins (64)	(#)	10 (25 ± 5x)	12 (21 ± 7x)	4 (18 ± 9x)	48 (33 ± 6x)	27 (27 ± 12x)
Cell division and growth (30)	(#)	23 (48 ± 9x)	23 (40 ± 7x)	14 (38 ± 10x)	25 (38 ± 8x)	12 (21 ± 14x)
tRNA metabolism (34)	(#)	27 (21 ± 4x)	26 (20 ± 5x)	12 (18 ± 9x)	28 (16 ± 4x)	6 (10 ± 5x)
F0F1 ATP synthase (7)	(#)	7 (46 ± 10x)	7 (45 ± 5x)	2 (15 ± 1x)	6 (42 ± 4x)	2 (23 ± 1x)
Complex I (11)	(#)	8 (37 ± 6x)	5 (35 ± 3x)	8 (24 ± 11x)	6 (21 ± 6x)	n.d.
Complex IV (4)	(#)	3 (39 ± 9x)	3 (37 ± 4x)	3 (26 ± 14x)	2 (43 ± 5x)	n.d.
Oxygen stress (11)	(#)	6 (30 ± 13x)	5 (22 ± 10)	4 (40 ± 8)	10 (46 ± 7x)	6 (18 ± 16x)
Nitrate reductase (3)	(#)	n.d.	1 (49 ± 3x)	1 (48 ± 2x)	3 (46 ± 6x)	n.d.
Nitrite reductase (1)	(#)	1 (44 ± 24x)	1 (44 ± 18x)	n.d.	1 (29 ± 32x)	n.d.
Nitric oxide reductase (1)	(#)	1 (44 ± 0x)	1 (44 ± 4x)	n.d.	1 (37 ± 8x)	n.d.
Nitrous oxide reductase (1)	(#)	1 (58 ± 3)	1 (49 ± 1x)	1 (60 ± 13x)	1 (66 ± 1x)	n.d.
Sulfide oxidation (13)	(#)	1 (51 ± 10x)	n.d.	n.d.	13 (36 ± 12x)	n.d.
Hydrogen oxidation (4)	(#)	n.d.	n.d.	n.d.	3 (27 ± 3x)	n.d.
Formate oxidation (5)	(#)	n.d.	n.d.	n.d.	5 (26 ± 5x)	n.d.
CO dehydrogenase (3)	(#)	3 (49 ± 16x)	n.d.	3 (40 ± 4x)	n.d.	n.d.
Calvin Cycle (3)	(#)	n.d.	n.d.	n.d.	3 (7 ± 1)	n.d.
Citric acid cycle (23)	(#)	16 (39 ± 7x)	15 (35 ± 5x)	13 (36 ± 8x)	17 (33 ± 5x)	n.d.
Sugar metabolism (24)	(#)	18 (44 ± 7x)	14 (35 ± 5x)	9 (28 ± 9x)	11 (29 ± 4x)	n.d.
Amino acid metabolism (44)	(#)	30 (28 ± 7x)	32 (26 ± 11x)	26 (26 ± 9x)	30 (26 ± 8x)	4 (11 ± 8x)
**Transporters**		**#Expressed proteins detected (peptide coverage)**				
Sugars (9)	(#)	6 (28 ± 7x)	3 (32 ± 7x)	6 (26 ± 5x)	2 (15 ± 16x)	n.d.
Aminoacids (7)	(#)	5 (34 ± 11x)	4 (59 ± 5x)	3 (19 ± 4x)	3 (46 ± 16x)	n.d.
Di/tricarboxylates (4)	(#)	4 (40 ± 14x)	4 (42 ± 10x)	3 (31 ± 15x)	2 (16 ± 17x)	n.d.
Glycine-betaine (4)	(#)	2 (36 ± 5x)	1 (46 ± 11x)	2 (30 ± 3x)	1 (44 ± 1x)	n.d.
Oligopeptides (3)	(#)	2 (31 ± 5x)	2 (32 ± 16x)	2 (33 ± 6x)	1 (19 ± 6x)	n.d.
Purines (1)	(#)	1 (55 ± 16x)	1 (65 ± 2x)	1 (61 ± 1x)	n.d.	n.d.
Acetate (1)	(#)	n.d.	n.d.	n.d.	1 (13 ± 1x)	n.d.
Urea (1)	(#)	n.d.	1 (42 ± 5x)	1 (30 ± 4x)	0	n.d.
Transporters total (29)	(#)	25	23	24	13	0

The abundance of the selected populations in the sediments was evaluated after screening four metagenomes sampled directly from the sediment for 16S rDNA genes and classification of the obtained 16S rDNA fragments. 104 out of 862 were classified as potential sulfur oxidizing Gammaproteobacteria (eight of these were >97% identical to the selected population, bin D), 35 as *Rhodobacterales* (ten of these were >97% identical to the selected populations, bin A-C) and 16 as BD1-5/SN-2 candidate phylum (see Table 4). For the latter reads the highest sequence identity to the selected population (bin E) was 93%.

**Table 4:** **Comparison of population abundances for the selected microbial community (based on metagenomics/FISH) and the sediment community composition *in situ* (based on sequencing reads encoding 16S rDNA genes)**.

**Taxon**	**Abundance *in situ* (%)**	**Abundance in the chemostat (%)**
Gammaproteobacteria	24.6^*^	19–25
Flavobacteria	10.2	n.d.
Cyanobacteria	10.1	n.d.
Deltaproteobacteria	9.0	n.d.
Eukaryoata	5.7	n.d.
Planctomycetes	4.8	n.d.
Actinobacteria	4.1	n.d.
Alphaproteobacteria	3.9	47–72
Acidobacteria	3.7	n.d.
Sphingobacteria	3.1	n.d.
Verrucomicrobia	2.8	n.d.
Cytophagia	1.9	n.d.
BD1-5/SN-2	1.9	1.4–11
Chloroflexi	1.4	n.d.
Others	12.8	n.d.

* 12.1% were affiliated with potential sulfur oxidizing gammaproteobacteria.

**Figure 5:**
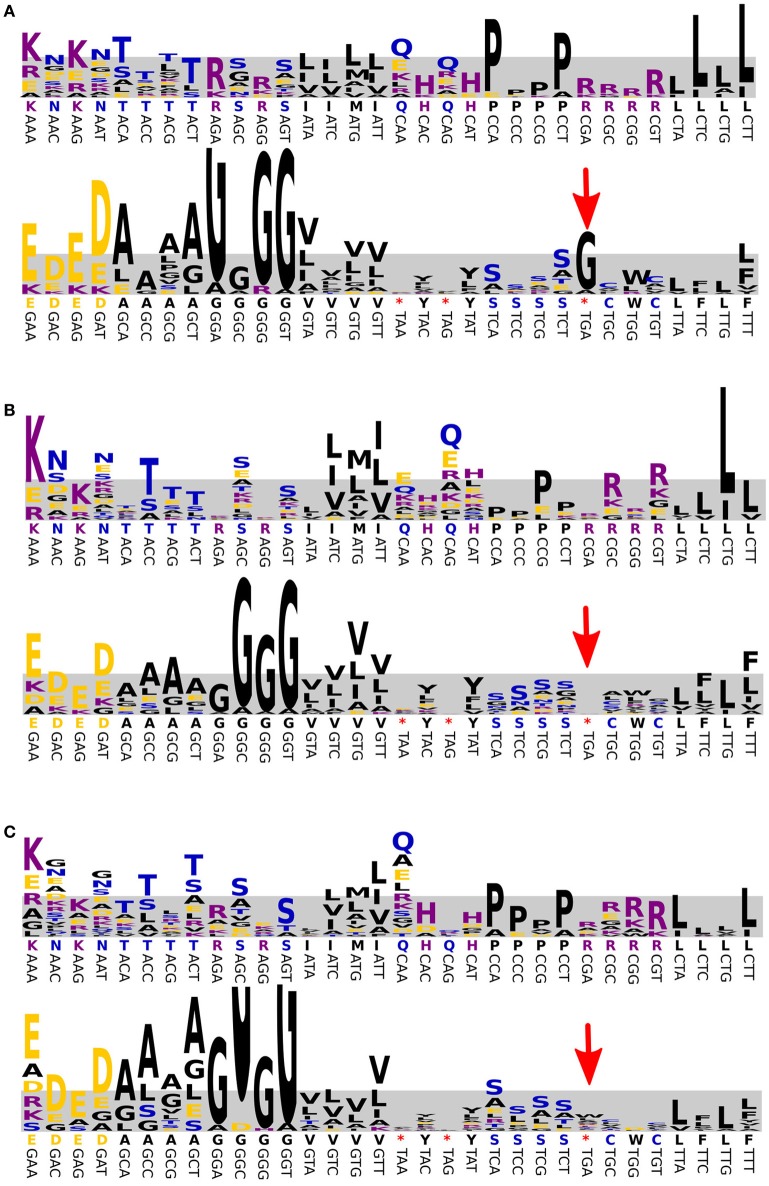
**Prediction of the genetic code used by the BD1-5/SN-2 population.** The results are shown for Bin E **(A)**, *E. coli* **(B)**, and Mycoplasma **(C)**. Directly above each codon, the canonical translation is shown (^*^indicates a stop codon). The frequency of amino acids observed in conserved positions of conserved proteins is visualized as a WebLogo, where the height of each letter is a measure for the observed frequency (most frequent amino acid shown at the top). The frequencies were normalized to the expected occurrence of the codon predicted from the percentage GC (shaded area). The UGA stop codon (red arrow) was correctly predicted for *E. coli* (stop) and *Mycoplasma* (tryptophan, W) and was predicted to be translated as glycine for the enriched BD1-5/SN-2 bacterium.

It is known that at least some bacteria of the BD1-5/SN2 clade and related bacteria (Gracilibacteria and clade SN1) are characterized by the use of an alternate genetic code (Wrighton et al., 2012; Campbell et al., 2013; Rinke et al., 2013). This is a very rare feature among prokaryotes and we first tested the likelihood that the enriched representative of this phylum also made use of a different code *in silico*. Indeed, the analysis of conserved gene sequences of bin E (Dutilh et al., 2011) suggested that the stop codon UGA was translated as glycine (Figure 5). To confirm the use of the alternate genetic code for this population, bin E was translated three times: UGA as stop, as tryptophan and as glycine. Proteomics measurements yielded a total of 27,449 non-redundant peptides from the predicted protein database with 1056 peptides contributed by the BD1-5/SN-2 population (SOM Tables 1, 3, 4). After applying a stringent cut-off of ±10 ppm mass accuracy on the resultant peptide-spectrum matches for BD1-5/SN-2, 820 peptides survived. Further interrogation of these peptides for their uniqueness within the three versions of BD1-5/SN-2 predicted proteome revealed that 97 of the peptides were unique to the BD1-5/SN-2 UGA-Gly database compared to just 3 peptides that were unique to the BD1-5/SN-2 UGA-Trp database (SOM Table 2). No peptides were found to be unique for the UGA Stop database. The remaining set of peptides was present in more than one version of the bin E database, rendering them not definitive for inferring codon usage. However, the occurrence of a large number of unique peptides corresponding to glycine versions of the database suggests that UGA preferentially translates to glycine in this species.

We further investigated whether the possible incidental translation of UGA as tryptophan was biologically real or an artifact of our method. Therefore, sequences of bin E were *in silico* translated with UGA coding for every standard amino acid and a new search was performed using previous database supplemented with these additional proteomes. Results were filtered at different stringencies (5–10 ppm mass accuracy on MS1 and a minimum of 2 filtered spectra). At the highest stringency, translation to tryptophan was still detected for a single peptide (compared to 40 for glycine). Interestingly, a single peptide was also matched to proteins from UGA coding for alanine, lysine and aspartate proteomes (SOM Table 2).

The five bins were annotated as individual provisional genome sequences that defined the metabolic potential of each of the corresponding populations. The proteomic analyses could now be used to infer which substrates might be used by the different populations. Good peptide coverage was obtained for proteins associated with each of the five bins, despite apparent differences in abundances of the populations. Total peptide counts for each bin agreed well with the abundances for each population estimated by metagenome analyses, except for the BD1-5/SN-2 population of bin E (Table 3).

The partitioning of metabolism over the different populations was inferred from the proteome. Table 3 shows the average peptide coverages and detected proteins for distinct subsystems for each of the bins and SOM Table 6 provides the data on all proteins detected. Proteomic analysis suggested that all populations except BD1-5/SN-2 (bin E) were competing for oxygen, whereas the denitrification pathway may have been characterized by a combination of competition and cross-feeding as can be seen from different steps of denitrification apparently performed by four different populations (bin A–D, Table 3). The expression of transporters, the enzymes involved in glycolysis, the citric acid cycle and respiratory complex I suggested that these four populations were also competing with each other for the supplied carbon substrates. The three *Rhodobacterales* (bin A–C) presumably used the substrates as energy and carbon source. In addition, the *Maritimibacter* population (bin A) may also have used sulfide as additional energy source, as shown by the expression of a sulfide dehydrogenase. The activity of some genes involved in the reversed citric acid cycle in the Gammaproteobacterium (bin D) suggested that this population may have mainly used the supplied organic molecules as a carbon source and not as an energy source; a partially reversed citric acid cycle is an indication for the interconversion of amino acids. It may have obtained energy for growth by oxidizing sulfide to sulfate, formate to carbon dioxide, and hydrogen to water. The expression of the Calvin cycle enzymes was also significant, indicating that it may have grown partially autotrophically. Based on the genomic and proteomic evidence, sulfide, hydrogen and formate did not appear to be produced by any of the major populations presented in Table 3. Their consumption hinted at the presence of other, sulfate reducing and fermentative populations that remained below the detection limit of our metagenome.

The expression of a monofunctional carbon monoxide dehydrogenase (Ni-Fe-CODH) by two of the *Rhodobacterales* (bins A and C) may indicate a presently unknown source of carbon monoxide. Turnover of this molecule is known to be important in many systems where it can be produced by photooxidation (Carpenter et al., 2012) but photooxidation could be excluded in our culture because it was always kept in the dark.

The expression of 27 ribosomal proteins and 12 proteins involved in cell division and growth was detected for the BD1-5/SN-2 population (bin E) and provided strong evidence for growth. However, we found very little evidence for most aspects of metabolism. Indeed, despite the fact that bin E was predicted to contain most of the sequence information present in the genome of this population (109/136 conserved proteins present, comparable to the *Roseobacter* in bin C, see Table 3), the enzymes of the citric acid cycle, glycolysis, and lipid biosynthesis were almost completely missing.

**Figure 6:**
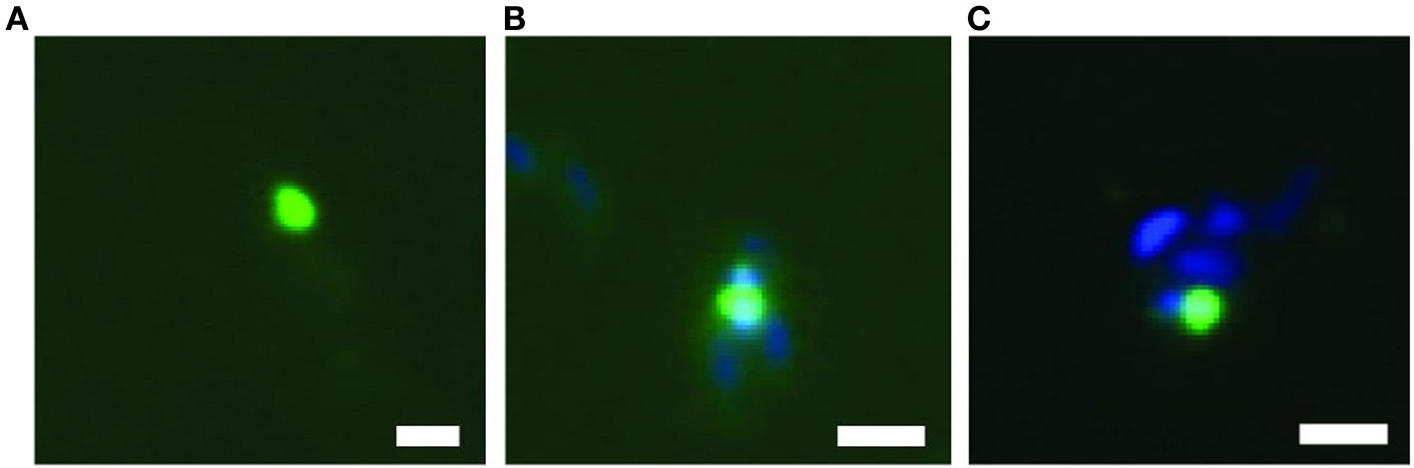
**Fluorescence *in situ* hybridization of bacteria of the BD1-5/SN-2 division, showing free living cells **(A)** and cells attached to other bacteria **(B,C)**.** Scale bar = 1 μm.

Finally, the relative abundances of the major populations as inferred from metagenomic and proteomic analyses were validated experimentally with fluorescence *in situ* hybridization (FISH) and microscopy (Tables 2, 3, Figure 6). The abundance of total Alphaproteobacteria (bin A–C) was 46.9%, of Gammaproteobacteria (bin D) 19% and of BD1-5/SN-2 (bin E) 1.4%. These numbers are consistent with the estimates derived from the metagenomic and proteomic data. The BD1-5/SN-2 cells were small and coccoid with a diameter of 0.3–0.5 μm. The cells were mainly free living but association to other cells was also frequently observed (Figure 6).

## Discussion

Microbial communities in convective tidal flat sediments are active in the remineralization of organic matter and denitrification (Kieskamp et al., 1991). Like many sediments they are characterized by a complex microbial community that defies current ecological approaches, for example to derive function-identity relationships or pinpoint community interactions (Lenk et al., 2011). In this study a laboratory chemostat was used to mimic the tidal flat conditions and select for a simplified tidal flat microbial community. The simplified community still contained representatives of populations that were abundant *in situ*. For most of these populations, the ecological functions and interactions with other community members could be provisionally resolved with proteomics. Ultimately, the functions as inferred from the proteome should be validated experimentally (e.g., isotope labeling, enzyme assays). However, the time needed for the experimental logistics and computational analyses of the metagenomic data was too long to enable the experimental follow-up required for further validation.

Although the extraction of microbes from the sediment led to a loss of microbial activity and induced changes in the microbial community composition, a detectable fraction of the *in situ* activity and community richness was preserved. Because we applied a selective force after the extraction procedure, representative extraction was not a prerequisite for our experiment as long as sufficient biodiversity and activity were preserved to allow for meaningful selection afterwards.

Thus, even though the conditions in the chemostat were very different from those in the sediment (e.g., the absence of sand), the *in situ* abundance of the selected populations was detectable. Apparently, the chemostat was still sufficiently close to the natural environment to select for some populations that were also successful *in situ*. The low concentrations of the substrates (micromolar range) in the chemostat might be one of the explanations for this succes, because selection of r-strategists (Andrews and Harris, 1986) could be prevented. Low substrate concentrations would be expected to select for a community of K-strategists; heterotrophic, facultatively aerobic denitrifiers. Indeed, after 23 days, the microbial community was dominated by three competing Alphaproteobacteria (bin A–C) that performed aerobic respiration and denitrification coupled to the oxidation of organic molecules as the carbon and energy source; two of these were members of the *Roseobacter* clade, the most abundant 16S rRNA gene lineage in marine ecosystems, and one population was related to *Maritimibacter alkaliphilus*, isolated from the Western Sargasso Sea (Atlantic Ocean) via high throughput culturing approaches (Lee et al., 2007; Thrash et al., 2010).

Interestingly, a population of potential sulfur oxidizing Gammaproteobacteria (bin D) was co-enriched with the Alphaproteobacteria. Proteomic analysis suggested that this population had a partially chemolithoautotrophic metabolism and used sulfide, formate and hydrogen as electron donors. Bacteria belonging to this clade of Gammaproteobacteria are very common at our sampling site (Lenk et al., 2011), in the oceans in general (e.g., SUP05; Walsh et al., 2009), and have also frequently been encountered as symbionts of marine invertebrates (e.g., Kleiner et al., 2012). The enriched organism was most closely related to the isolates *Sedimenticola selenatireducens* (Narasingarao and Häggblom, 2006), *Thiotaurens thiomutagens* (Flood, 2010), and *Dechloromarinus chlorophilus* (Coates and Achenbach, 2004; Clark et al., 2013). Its 16S rDNA sequence was 94% identical to a sequence previously detected in Janssand sediments (HQ191061, Lenk et al., 2011) and 88% identical to the SUP05 16S rDNA genes. The active expression of a complete sulfide oxidation pathway (including flavocytochrome c sulfide dehydrogenase, dissimilatory sulfite reductase, adenylsulfate reductase, sulfate adenyl transferase) by the potential sulfur oxidizing Gammaproteobacterium in the chemostat suggested the activity of a so called “cryptic” sulfur cycle, whereby sulfate reduction and sulfide re-oxidation are so tightly coupled that sulfide does not accumulate (Canfield et al., 2010). However, no sulfide was detected and no evidence for the presence of sulfate reducing bacteria was found. Therefore, it is perhaps more likely that the sulfide oxidation pathway was expressed constitutively, without an active function in the chemostat enrichment. The activity of the Calvin cycle (e.g., expression of ribulose bisphosphate carboxylase, phosporibulokinase) suggested a (partially) autotrophic lifestyle. Even though organic compounds (glucose, aminoacids) were continuously supplied to the chemostat, microbial competition for these substrates led to low actual substrate concentrations in the chemostat. It is possible that the selected population of Gammaproteobacteria also made use of the energy conserved from formate and hydrogen oxidation to assimilate CO_2_ as an additional carbon source. This was apparent from the active expression of formate dehydrogenase and a nickel/iron uptake hydrogenase (see also Table 3). These physiological interpretations where compared to what is known for related, cultivated bacteria: A provisional genome sequence is available for *Dechloromarinus chlorophilus* (Clark et al., 2013) and the genome of this bacterium also encodes genes for formate dehydrogenase, a nickel/iron uptake hydrogenase, key Calvin cycle enzymes (ribulose bisphosphate carboxylase and phosphoribulokinase) but only a single gene of the sulfide oxidation pathway (*dsrB*). For *T. thiomutagens* a genome sequence is not available but the physiology of this organism is consistent with the proteomic results presented here. Both the activity of the Calvin cycle and sulfur oxidation were previously shown to be detectable for potential sulfur oxidizing Gammaproteobacteria in different marine habitats (Lavik et al., 2009; Walsh et al., 2009; Canfield et al., 2010; Stewart, 2011).

Candidate division BD1-5/SN-2 and the closely related candidate phyla SR1 are very common components of microbial communities in very different habitats such as the human gut, the oral cavity (Campbell et al., 2013), subsurface aquifers (Wrighton et al., 2012) and marine sediments. At our sampling site almost 2% of all 16S rRNA genes identified in 4 different metagenomes belonged to this division. Members of BD1-5/SN-2 have not been cultivated so far but a fermentative, possibly sulfur reducing lifestyle has been inferred from genomes reconstructed from metagenomic data (Wrighton et al., 2012) and single cell genomes (Campbell et al., 2013). Their abundance in the community selected here (bin E) was between 1.4 and 5%, as shown by FISH/proteomics and metagenomics, respectively. The relatively short contigs in bin E and the significant degree of internal overlap in this bin suggested that the enriched population was not clonal; even though only a single 16S rRNA gene sequence was recovered (assembly and iterative read mapping produced identical sequences). The cross-assembly of multiple, closely related populations in bin E led to a considerable degree of redundancy and an overestimation of the genome size, which is probably still significantly lower than the 2.1 Mb estimated in Table 3.

Both *in silico* analysis and proteomics showed very strong evidence for the use of an alternate genetic code by this bacterial division. As was previously described for the related division SR1 and Gracilibacteria, the opal stop codon, UGA, was found to be translated as glycine (Campbell et al., 2013; Rinke et al., 2013). Alternate genetic codes are relatively rare among bacteria, but also translation of UGA as tryptophan is known in multiple organisms (Yamao et al., 1985; McCutcheon et al., 2009).

In depth investigation of the possible translations of UGA in bin E by proteomics also showed that low levels of “mistranslation” may occur: a single peptide was detected for tryptophan, alanine, aspartate and lysine. Such mistranslations may actually be a prerequisite for the evolution of alternate genetic codes as the one reported here.

The BD1-5/SN-2 genomic dataset in bin E encodes very little recognizable metabolic capacity. Very few genes were detected that encoded enzymes involved in respiration. Genes encoding enzymes involved in the citric acid cycle, glycolysis, amino acid metabolism, and lipid metabolism were almost completely absent, a finding that parallels the prior results of Wrighton et al. (2012) and Rinke et al. (2013). The only enzyme complex that was clearly expressed was the F0F1 ATP synthase. The apparent lack of encoded metabolic capacity could not easily be explained by annotation problems because the genes encoding cell biological processes (DNA replication, transcription, translation and cell division) were detected without problems (and were also actively expressed). Based on the lack of metabolism, a highly syntrophic or parasitic lifestyle of the enriched BD1-5/SN-2 bacterium seems to be the only possible explanation for these findings. However, fluorescence *in situ* hybridization provided only limited evidence for the association of BD1-5/SN-2 with other cells (Figure 6); most cells appeared to be free living. The identification of the ecological niche of the microbes that constitute the BD1-5/SN-2 phylum will be an exciting avenue for future investigations.

The relative abundances of potential sulfur oxidizing Gammaproteobacteria (bin D) and BD1-5/SN-2 (bin E) in the selected microbial community were close to the *in situ* abundances (Table 4). Other populations that were abundant *in situ* were not selected: Flavobacteria, Eukaryota, Cyanobacteria and Deltaproteobacteria. Flavobacteria are known as polymer degraders (Kirchman, 2002) and their selection was not expected because the substrates were supplied as monomers. Cyanobacteria were excluded because the incubation was performed in the dark and Eukaryota were removed from the inoculum by the addition of cyclohexamide. Deltaproteobacteria (e.g., sulfate reducers) might be more successful *in situ* than in the incubation because of the presence of oxygen and nitrate gradients *in situ* leading to anoxic micro-niches which were absent in the incubation. As a consequence, most of the supplied substrates were consumed by Alphaproteobacteria instead and these came to dominate the selected community.

In conclusion, in this study we engineered specific aspects of the marine tidal sediment habitat in a laboratory continuous culture incubation. In this incubation, the environmental conditions selected for specific populations of the natural sediment community and allowed us to study these populations in the context of a simplified microbial community. Metagenomic and proteomic approaches provided the first experimental evidence for the translation of the stop codon UGA as glycine by a member of the BD1-5/SN-2 phylum and also provided hints about the niche partitioning of Alphaproteobacteria and potential sulfur oxidizing Gammaproteobacteria with regard to carbon metabolism. In future studies, similar approaches could be used to address key problems in microbial ecology, such as the cryptic sulfur cycle, community dynamics resulting from viral predation, antagonistic interactions between baceria, cross feeding and competition. Such ecological phenomena cannot be studied with isolated strains and although direct observation of natural ecosystems may also yield results, that approach is much more challenging because of the higher complexity and the lack of experimental control.

## Author contributions

Anna Hanke and Emmo Hamann performed sampling and cultivation with support from Jeanine S. Geelhoed and Marc Strous. Anna Hanke performed FISH with support from Sabine Lenk. Halina E. Tegetmeyer performed DNA sequencing and assembly. Beate Kraft, Anna Hanke, and Theresa Hargesheimer performed the benchmarking of the sediment extraction. Controllers were designed and tested by Marc Strous, Kofi Makinwa, Rong Wu, and Volker Meyer and assembled by Harald Osmers. Ritin Sharma and Robert L. Hettich performed proteomic analyses. The manuscript was written by Anna Hanke and Marc Strous with support from all other authors.

### Conflict of interest statement

The authors declare that the research was conducted in the absence of any commercial or financial relationships that could be construed as a potential conflict of interest.
